# Evaluation of the population structure and genetic diversity of *Plasmodium falciparum* in southern China

**DOI:** 10.1186/s12936-015-0786-0

**Published:** 2015-07-22

**Authors:** Guiying Wei, Lili Zhang, He Yan, Yuemeng Zhao, Jingying Hu, Weiqing Pan

**Affiliations:** Institute for Infectious Diseases and Vaccine Development, Tongji University School of Medicine, 1239 Siping Road, Shanghai, 200092 China; Department of Tropical Infectious Diseases, Second Military Medical University, 800 Xiang Yin Road, Shanghai, 200433 China

**Keywords:** Malaria, *Plasmodium falciparum*, Genetic diversity, Molecular markers, Microsatellite

## Abstract

**Background:**

Yunnan and Hainan provinces are the two major endemic regions for *Plasmodiumfalciparum* malaria in China. However, few studies have investigated the characteristics of this parasite. Therefore, this study aimed to evaluate the genetic diversity and population structure of *P. falciparum* to predict the geographic origin of falciparum malaria.

**Methods:**

Thirteen highly polymorphic microsatellite loci were studied to estimate the genetic diversity and population structure of 425 *P. falciparum* isolates obtained from blood samples collected from Yunnan and Hainan provinces of South China. The isolates were analysed for genetic diversity, linkage disequilibrium, and population structure. The parasite populations were clustered into two subgroups (i.e., Yunnan and Hainan) and a classification algorithm was used to identify molecular markers for classifying the *P. falciparum* populations.

**Results:**

All 13 microsatellite loci were highly polymorphic, with the number of alleles per locus varying from 5 to 20. The mean expected heterozygosity (*He*) in Yunnan and Hainan was 0.766 ± 0.036 and 0.677 ± 0.039, respectively, revealing a moderate high level of genetic diversity. Significant linkage disequilibrium was found for some regions of Yunnan (Lazan county and Xishuangbanna region) and Hainan (Dongfang city and Sanya city) province. According to the classification algorithm, a combination of three microsatellites could be used as a discriminatory marker to identify the origin of *P. falciparum* isolates.

**Conclusions:**

The results on the genetic structure of *P. falciparum* populations from South China provide a basis for developing a genetic marker-based tool to trace the source of the parasite infections and consequently improve malaria control and elimination strategies.

**Electronic supplementary material:**

The online version of this article (doi:10.1186/s12936-015-0786-0) contains supplementary material, which is available to authorized users.

## Background

Malaria, a mosquito-borne infectious disease, remains a serious public health problem in many tropical and subtropical countries, affecting millions of individuals every year. Among the five variants of malaria affecting humans, the disease caused by *Plasmodium falciparum* (falciparum malaria) is the most severe form and can be fatal [1]. In southern China, Yunnan and Hainan provinces are the two major endemic regions for falciparum malaria [2–5].

Yunnan province located in southern continent of China, with more than 80% of the population in this province at risk of malaria infection [6] and in the past decade, Yunnan ranked No.1 in the country in terms of the number of cases [2–4]. Hainan province is on the southern coast of China, and account for up to 46% of the annual endemic *P. falciparum* malaria cases over the past decade [4]. It is emergent to take effective measures to control and eliminate *P. falciparum* in southern China.

Although the malaria burden in these regions has significantly decreased of late, imported malaria cases have been increasing and pose a major challenge for malaria control and elimination programmes [3, 7, 8]. Therefore, the development of molecular tools to identify the source of imported malaria parasites and trace the migration of the local parasites has become extremely important. Molecular genotyping techniques have been used to analyse the genetic diversity, transmission dynamics, and population structure of *P. falciparum* field isolates. Early molecular studies focused on loci encoding parasite surface antigens, such as merozoite surface protein 1 (MSP1), merozoite surface protein 2 (MSP2), and glutamate-rich protein (Glurp) [9–11], circumsporozoite surface protein (CSP) [12], and apical membrane antigen 1 (AMA1) [13]. These loci are often under strong immune selection pressure [14, 15], the genotyping results provided by these markers can potentially lead to a masked and distorted view of the population structure and transmission patterns. Currently, simple sequence repeats of microsatellite loci, considered “selectively neutral” loci, are powerful markers for population genetic studies. In the *P. falciparum* genome, microsatellite loci are extraordinarily abundant, occurring at an average rate of one in every 2–3 kb of the *P. falciparum* sequence and predominantly as [TA]_n_, [T]_n_, and [TAA]_n_ repeats [16, 17].

Since Anderson et al. first described protocols for the characterization of 12 microsatellite markers from samples infected with *P. falciparum*, microsatellite markers have been used to study the genetic diversity and population structure of *P. falciparum* populations in many several countries worldwide [18–26]. On a global scale, Anderson et al. [19] adapted 12 trinucleotide repeat loci to measure genetic diversity using 465 *P. falciparum*-infected blood samples collected from different regions in Africa, South America, and Southeast Asia. The study revealed a spectrum of population structures for *P. falciparum*: in regions with low transmission, strong linkage disequilibrium (LD), low diversity, and extensive population differentiation were observed, while in regions with high transmission, weak LD, high diversity, and low levels of differentiation were observed [19]. In other studies, microsatellite markers were used to analyse the population genetics of *P. falciparum* samples collected from a single region or country, including Thailand [20], Philippines [21], Malaysian Borneo [22], Papua New Guinea [23], Western Kenya [24, 25] and the Republic of the Congo [26]. In China, molecular biological studies on drug-resistant malaria were recently reported [27, 28], although studies using neutral microsatellite markers to analyse the basic genetic diversity of the parasite are limited.

In the present study, 13 highly polymorphic microsatellite loci were analysed to estimate the genetic diversity and population structure of *P. falciparum* in Yunnan and Hainan provinces of South China. The relationship between the two populations was also evaluated and a genetic database of the parasite from South China was established. Last, a classification algorithm was used to select effective microsatellite marker sets to classify the parasite populations from the two regions.

## Methods

### *Plasmodium falciparum* sample collection and study sites

**Figure 1:**
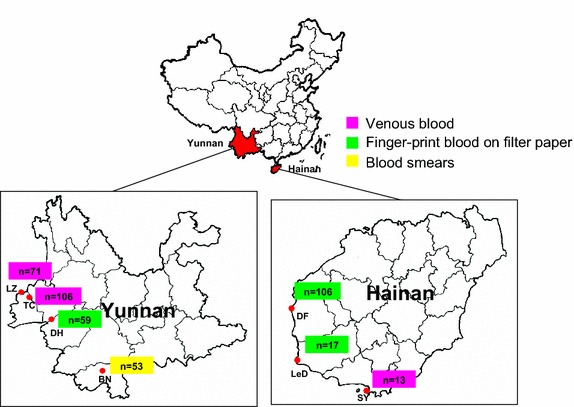
A schematic map showing the distribution of sampling sites for *Plasmodium falciparum.*

In total, 425 *P. falciparum*-infected blood samples were collected from seven regions in China from 2004 to 2008, including three regions in Hainan province, three in Yunnan province, and a town located at the border of China and Myanmar (Figure 1; Table 1). The study locations, sample size (n), types of patient blood collected and years of collection were as follows: Hainan province (N = 136), with venous blood samples treated with EDTA from Sanya city (SY, n = 13) collected in 2005,2007; finger-prick blood spot samples collected on filter paper from Dongfang city (DF, n = 106) collected in 2004, 2007, and 2008; and Ledong county (LeD, n = 17) collected in 2004; and Yunnan province (N = 289), with finger-prick blood spot samples collected on filter paper from Dehong region (DH, n = 59) collected in 2007, venous blood samples treated with EDTA from Tengchong county (TC, n = 106) collected in 2006 and Lazan county (LZ, n = 71) collected in 2006, 2007; and blood smears from Xishuangbanna region (BN, n = 53) collected in 2006. Most samples (n = 349) were previously genotyped as part of a survey on pyrimethamine resistance in South China [27].

**Table 1:** Number of samples collected: single- and multiple-clone infections in *Plasmodium falciparum* populations

Site	Number of samples collected in the year					N	n	Single	Multiple (%)
	2004	2005	2006	2007	2008				
Yunnan									
LZ	–	–	67	4	–	71	66	58	8 (12%)
DH	–	–	–	59	–	59	49	41	8 (16%)
TC	–	–	106	–	–	106	93	72	21 (23%)
BN	–	–	53	–	–	53	29	26	3 (10%)
Hainan									
DF	22	–	–	36	48	106	93	83	10 (11%)
LeD	17	–	–	–	–	17	15	15	0 (0%)
SY	–	8	–	5	–	13	13	11	2 (15%)
Total	39	8	226	104	48	425	358	306	52 (15%)

*N* number of collected isolates, *n* number of isolates successfully genotyped at all 13 microsatellite loci, *single* number of single infections detected by PCR amplification, *multiple* number of samples with multiple infections exhibiting at least one allele at one locus.

All samples were collected from symptomatic patients with blood smears that tested positive for *P. falciparum* at the local Centre for Disease Control and Prevention (CDC). Prior informed consent was obtained from the patients, and the study was reviewed and approved by the Ethical Review Board of the Second Military University, China.

### DNA extraction and microsatellite genotyping

Genomic DNA of the parasite from blood samples, including venous blood samples, finger-prick blood samples on filter papers, and blood smears, was extracted and purified using the QIAamp DNA Blood Mini Kit (QIAGEN) according to the manufacturer’s instructions. The extracted DNA samples were stored at −20°C before use. In addition to the field samples, genomic DNA from the *P. falciparum* laboratory-adapted strain 3D7 was extracted and purified for use as a positive reference.

A total of 13 trinucleotide repeat microsatellite loci distributed throughout the genome of *P. falciparum* was studied: TA1 (Chr6), Polyα (Chr4), PfPK2 (Chr12), TA81 (Chr5), TA109 (Chr6), TA42 (Chr5), TA60 (Chr13), TA87 (Chr6), ARA2 (Chr11), 2490 (Chr10), Pfg377 (Chr12), C1M8 (Chr1), and B5M2 (Chr7). The first 12 loci were previously used as putatively neutral microsatellite markers for population genetic studies [18, 19, 29], and the last locus (B5M2) was discovered during a selective sweep study of resistance induced by *pfcrt*, which is located 115 kb upstream of the chloroquine resistance transporter gene (data not published).

The microsatellites were amplified by a two-step semi-nested PCR strategy, and the reaction conditions followed the protocol of Anderson et al. [18]. The positive PCR products were run using the QIAxcel capillary electrophoresis platform with a high-resolution gel cartridge (Qiagen), and the allele length and peak heights from each locus were analysed and visualized using the Bio-Calculator software (Qiagen). Because the blood stages of *P. falciparum* are haplotypic, all isolates exhibiting single infection (each isolate exhibiting only one allele for all 13 microsatellite loci) were included in subsequent analyses. Data from samples with poor amplification and samples exhibiting multiple infections on genotyping were excluded. As described previously, in a given isolate, minor peaks taller than one-third the height of the predominant allele for each locus were considered to represent multiple alleles per locus [19]. Multiple infections were assessed by the presence of multiple alleles at any of the surveyed microsatellite loci.

### Population genetic analysis

#### Genetic diversity

Population genetics were preferably assessed only in samples with single infections, because the use of samples with multiple infections could result in bias. To measure the genetic diversity, the number of haplotypes (h), number of different alleles (*Na*), number of effective alleles (*Ne*), number of private alleles (*Np*), and the expected heterozygosity (*He*) were evaluated using the Excel plug-in software GenAlex 6.5 [30, 31]. *He* was calculated using the following formula: [n/(n − 1)] (1 − ∑P_*i*_^2^), where n is the number of sampled infections and P_*i*_ is the frequency of the *i*th allele.

#### Analysis of multilocus linkage disequilibrium

Multilocus LD, defined as the nonrandom association among alleles from all 13 screened loci, was analysed using the program LIAN version 3.5 [32]. The standardized index of association (I_A_^S^) was used to assess multilocus LD in each *P. falciparum* population from Yunnan and Hainan provinces. The I_A_^S^ values were calculated using the formula (V_D_/Ve − 1)/(r − 1) with permutation testing of the null hypothesis of I_A_^S^ considered as 0 (complete LD), where V_D_ represents the observed variance in mismatch values, Ve represents the expected variance in mismatch values, and r represents the number of analysed loci. The significance of the I_A_^S^ values was tested using the Monte Carlo method.

#### Dendrogram of pairwise inter-population genetic distance analysis

The pairwise Fst indices was employed to quantify the genetic distance between pairs of populations, and was calculated using Arlequin 3.1 [33]. The genetic distance matrix was then used to construct a dendrogram of inter-population clustering by implemented the program MEGA4 [34].

#### Population structure

The program STRUCTURE version 2.3.4 [35, 36] was used to test whether individuals clustered according to geographic origin, employing a Bayesian approach to identify the number of clusters (K) in the dataset without any prior information on the origin of the population. The range of possible genetic clusters was run from K = 1–14 using the admixture ancestry model, with the runs for K = 2–10 repeated 10 times and those for K = 11–14 repeated six times. Each run was implemented with a burn-in period of 50,000 iterations and 100,000 Markov Chain Monte Carlo replications. Then, ΔK was used to determine the optimal K according to the method described by Evanno et al. [37]. The ΔK value corresponded with the second order rate of change in the likelihood function with respect to K. In order to further represent the geographical distribution and provide an alternative view of substructuring, a median-joining algorithm analysis [38] was conducted by using 13 locus microsatellite haplotypes through the program Network version 4.6.1.2 [39].

#### Identification of genetic markers for local populations

To identify the most reliable discriminatory microsatellite marker sets that would best differentiate *P. falciparum* isolates between Yunnan and Hainan provinces, data were analysed using Weka software (version 3.7.1) [40]. The classification algorithm used with Weka 3.7.1 was a support vector machine (SVM) algorithm known as LibSVM [41], with standard parameters and tenfold cross-validation for accuracy estimation.

## Results

Of the total of 425 blood samples, 358 samples were successfully genotyped at all 13 microsatellites (Additional file 1). Of these, 306 samples exhibited single alleles per locus, indicating single infection, while the remaining 52 showed multiple alleles per at least one locus, implying multiple infections (Table 1). In the case of multiple infections, the proportions ranged from 10% in BN to 23% in TC. While, in the LeD population, all 17 samples genotyped across all 13 loci displayed as single infection. Though proportion of multiple infections quiet different in the seven populations, there are no significant differences among the populations (Fisher’s exact test, p = 0.176). The samples with multiple infections were thus excluded from the dataset, and the 306 samples with single infection were recruited for population genetic analysis; their allele frequencies per locus are shown in Additional file 2.

### Genetic diversity of microsatellites

A total of 157 alleles were identified from the 306 isolates through analysis of the 13 microsatellites (Additional files 2, 3). All 13 microsatellites were high polymorphic, with the number of alleles for each microsatellite varying from 5 to 20. The average number of alleles per locus was 12.154 ± 1.285. *He* was used as a measure of variation. The *H*e values of the microsatellites varied from 0.58 to 0.91, and the mean heterozygosity of the loci was 0.769 ± 0.031, indicating moderate diversity.

Next, comparison of the overall genetic diversity of *P. falciparum* isolates from different regions. As shown in Table 2, the isolates from Yunnan province showed extremely high diversity in terms of the mean *Na* (11.69 ± 1.22), *Np* (30), and mean *He* (0.766 ± 0.036) compared with the isolates from Hainan, who showed corresponding values of 6.923 ± 0.923, 4, and 0.677 ± 0.039, respectively.

**Table 2:** Genetic diversity parameters for the sampled *Plasmodium falciparum* populations

Population	N	Np	Na ± SE	Ne ± SE	He ± SE
Yunnan	197	30	11.692 ± 1.216	5.448 ± 0.767	0.766 ± 0.036
LZ	58	3	8.385 ± 1.083	4.513 ± 0.594	0.726 ± 0.047
DH	41	11	8.308 ± 0.763	4.466 ± 0.546	0.726 ± 0.053
TC	72	8	9.000 ± 1.092	4.831 ± 0.786	0.698 ± 0.064
BN	26	8	5.923 ± 0.512	3.695 ± 0.401	0.702 ± 0.049
Hainan	109	4	6.923 ± 0.923	3.622 ± 0.446	0.677 ± 0.039
DF	83	1	5.923 ± 0.738	3.148 ± 0.325	0.642 ± 0.040
LeD	15	2	4.308 ± 0.398	2.972 ± 0.275	0.673 ± 0.035
SY	11	1	4.000 ± 0.376	2.746 ± 0.280	0.660 ± 0.034
Total	306	34	12.154 ± 1.285	5.376 ± 0.753	0.769 ± 0.031

*n* number of sampled individuals, *Na* number of different alleles, *Ne* number of effective alleles, *He* expected heterozygosity, *Np* number of private alleles.

Comparison of the diversity of the 13 microsatellites for the isolates from the seven regions showed the highest mean *Na* (9.000 ± 1.092) for the isolates from TC, and the highest mean *He* for the isolates from LZ (0.726 ± 0.047) and DH (0.726 ± 0.053).

### Multilocus linkage disequilibrium

LD was measured first for all complete haplotypes, including single and multiple infections (n = 358), and subsequently tested only for single infections by removing alleles from multiple infections (n = 306). Because all haplotypes in the dataset were unique, it was not necessary to measure LD among unique haplotypes, as observed in a previous study [23]. A strong and significant LD (p < 0.0001) was found within the whole dataset (Table 3). The analysis of all infection haplotypes shows that significant linkage disequilibrium was identified in four populations as LZ, BN, DF and SY, and the degree of LD ranged from 0.027 (LZ, p < 0.0001) to 0.117 (SY, p < 0.0001), while there was no evidence for LD in DH, TC, or LeD. Similar results were obtained when only single infections were analysed. After a correlation analysis, a weak negative correlation was found between the proportion of multiple infections and the degree of LD (Spearman’s R = −0.357), and the correlation was not significant.

**Table 3:** Multilocus linkage disequilibrium (the standardized index of association I_A_^S^) in *Plasmodium falciparum* populations in South China

Population	All Infections			Single Clones		
	N	I_A_^S^	P value	N	I_A_^S^	P-value
Yunnan	237	0.0147	<0.0001	197	0.0182	<0.0001
LZ	66	0.0266	<0.0001	58	0.0324	<0.0001
DH	49	−0.005	0.882	41	−0.001	0.582
TC	93	0.0009	0.397	72	0.0003	0.452
BN	29	0.0659	<0.0001	26	0.0829	<0.0001
Hainan	121	0.0843	<0.0001	109	0.0791	<0.0001
DF	93	0.1077	<0.0001	83	0.105	<0.0001
LeD	15	0.005	0.337	15	0.005	0.335
SY	13	0.1171	<0.0001	11	0.1295	<0.0001
Total	358	0.0233	<0.0001	306	0.0245	<0.0001

### Dendrogram of pairwise inter-population genetic distance analysis

**Figure 2:**
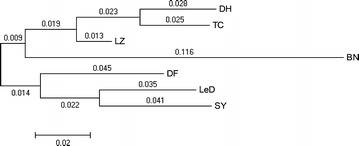
Dendrogram of pairwise inter-population genetic distance analysis.

Pairwise comporations of genetic distance between all *P. falciparum* populations studied were shown in Table 4. The Fst values ranged from 0.035 (between LZ and TC populations) to 0.214 (between BN and SY populations). The genetic distance varied from the four study sites of Yunnan province. In particular, higher level of differentiation was demonstrated with Fst between BN and the other three sites (>0.140). Meanwhile, pairwise Fst values between DF and LeD (0.105), and DF and SY (0.104) were similar, but a little low between LeD and SY population (0.076). The dendrogram based on the pairwise Fst matrix showing a separate clustering of populations from Yunnan and Hainan (Figure 2). The four study sites of Yunnan province belong to a cluster, while the three study sites of Hainan province were assigned to another cluster. It can providing a simply evidence of geographical sub-population structure.

**Table 4:** Pairwise genetic distance (Fst) between Plasmodium falciparum populations from seven sites of Southern China

Site, population	Yunnan Province				Hainan Province		
	LZ	DH	TC	BN	DF	LeD	SY
Yunnan Province							
LZ	–						
DH	0.086	–					
TC	0.039	0.053	–				
BN	0.168	0.146	0.182	–			
Hainan Province							
DF	0.119	0.157	0.159	0.164	–		
LeD	0.090	0.135	0.147	0.204	0.105	–	
SY	0.099	0.168	0.153	0.214	0.104	0.076	–

### Population structure

**Figure 3:**
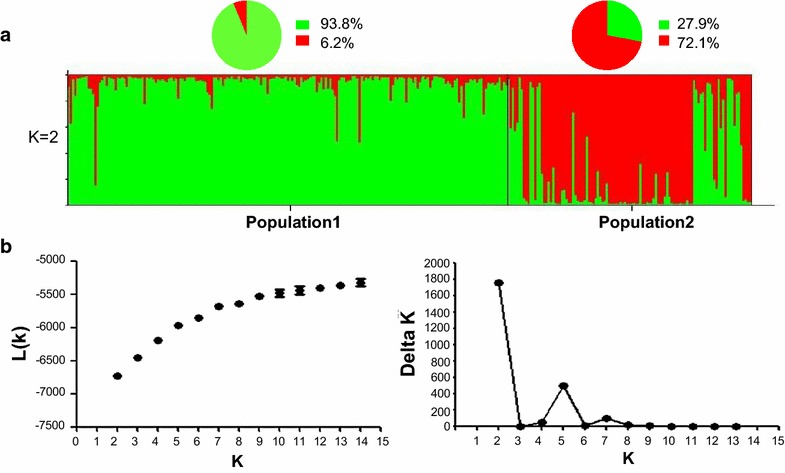
Bayesian cluster analysis using the STRUCTURE program: results for K = 2. **a** Graphical representation of the dataset for the most likely K (K = 2), where each *colour* corresponds to a suggested cluster and each individual is represented by a *line*. **b** *Graphs* show the mean L (K) (±SD) for each value of K from 2 to 15. ΔK was calculated according to the method of Evanno et al. [37].

Population structure studies were used to identify and characterize the *P. falciparum* populations according to the allele frequencies at each locus. According to the 13 microsatellite data set, cluster analysis showed that the primary peak of ΔK was observed at K = 2 (Figure 3), suggesting that the entire *P. falciparum* population from South China could be divided into two subgroups (Pop1 and Pop2). In Pop1, 93.8% members from the Yunnan–Myanmar border regions and 6.2% members from Hainan province were clustered together. In contrast, 72.1% members from Hainan province clustered with 27.9% members from the Yunnan province in Pop2. Hence, the parasite populations were clustered based on their geographic origins. When the number of subgroups was increased from K = 2 to K = 5 (Additional file 4), the members in Pop1 could be further assigned to different sub-subgroups, particularly those from LZ, TC, and DH. The members from BN varied from the LZ, TC, and DH members; most members were assigned to different sub-subgroups. In contrast, the members in Pop2 were always assigned to two clusters. A majority of the *P. falciparum* isolates collected from 2004 to 2007, irrespective of belonging to DF, LeD, or SY, was clustered together; however, the isolates collected in 2008 from DF were assigned to another subgroup.

**Figure 4:**
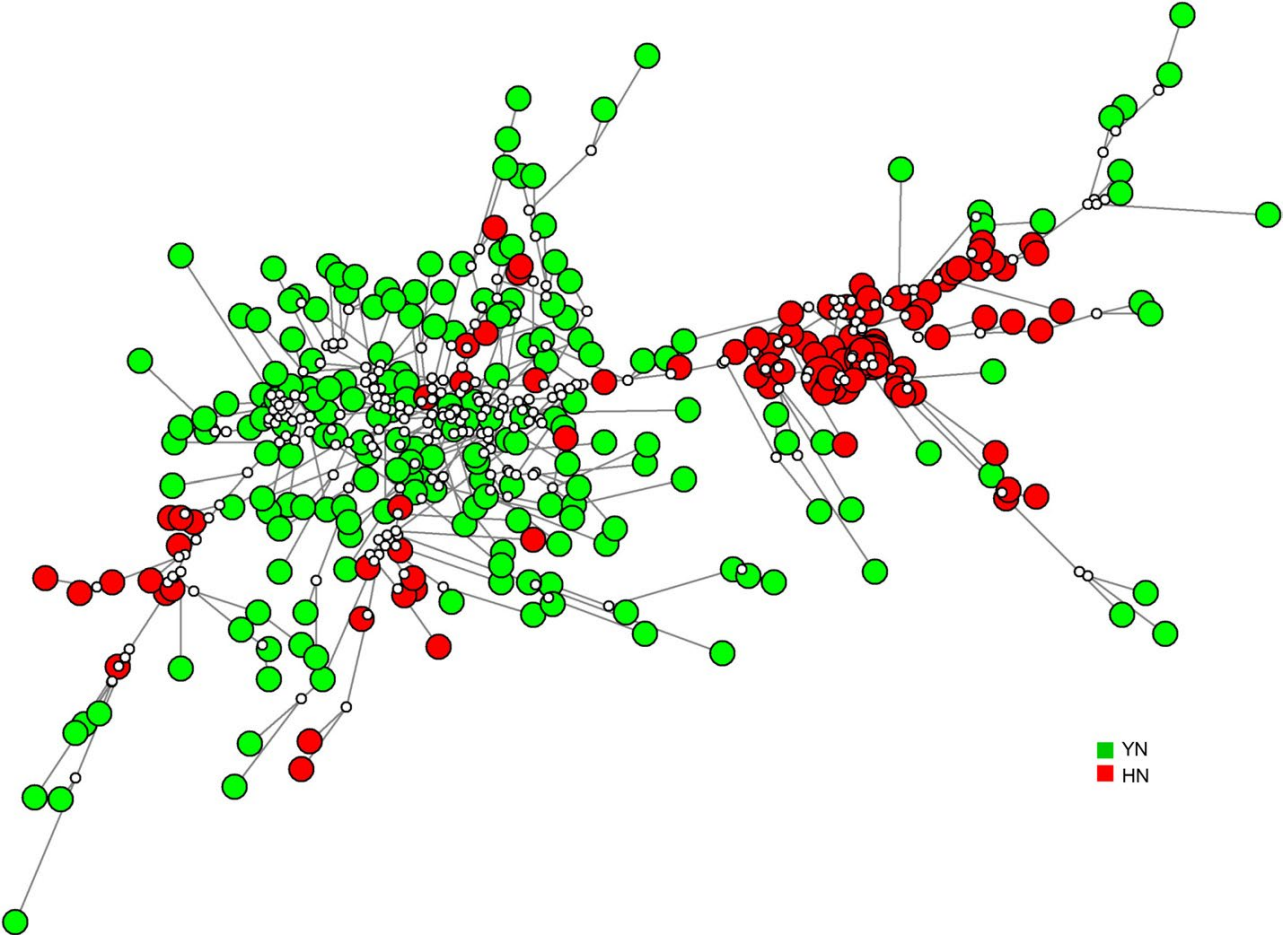
Network analysis of microsatellite haplotypes from southern China. Each *coloured circle* represents a haplotype, *green* Yunnan (YN), *red* Hainan (HN). *White dots* connecting haplotypes within the network are hypothetical median vectors generated by the software.

To further confirm the above results for the population structure, the 13 locus microsatellite data set was analysed using the Network. As shown in Figure 4, all haplotypes were constructed into two major independent groups. Compared with structure analyses, the results of network analysis were consistent with the patterns at K = 2, and supported the conclusion that *P. falciparum* populations are clearly clustered according to their geographic origin. In network analysis, central types are usually possible ancestors, and peripheral types are descendants [42]. It should be noted that, a few haplotypes from Hainan migrated into the Yunnan group. While, some Yunnan haplotypes were found in the Hainan group, and all were assigned peripheral. The results suggesting that, both Yunnan and Hainan with an independent ancestor, and some epidemiology reasons such as transmission, leading the migration of haplotypes.

### Identification of genetic markers for local populations

By combining and recombining the microsatellite loci into different marker sets using Weka 3.7.1, we gradually obtained marker sets with combinations of one, two, three, or four loci, and these could differentiate *P. falciparum* isolates between Yunnan and Hainan provinces with high accuracy (Additional file 5). The LibSVM algorithm revealed that when three microsatellite loci, namely TA1, Pfg377 and B5M2 were used as variables to classify *P. falciparum* isolates, the correctly classified instances were 94.118%, and the rates of correct classification were 97 and 89% for Yunnan and Hainan provinces (Table 5), respectively. While 2490, Pfg377, and B5M2 were used as variables, the correctly classified instances were 92.157% and the correct classification rates were 92.4 and 91.7% for Yunan and Hainan provinces, respectively.

**Table 5:** Selection of the most reliable marker set to identify the origin of *Plasmodium falciparum* isolates

Marker set				Marker set			
TA1, Pfg377 & B5M2				2490, Pfg377 & B5M2			
Total number of instances 306				Total number of instances 306			
Correctly classified instances (288) 94.118%				Correctly classified instances (282) 92.157%			

Detailed accuracy by class				Detailed accuracy by class			
TP Rate	FP Rate	Precision	Class	TP Rate	FP Rate	Precision	Class
0.97	0.11	0.941	Yunnan	0.924	0.083	0.953	Yunnan
0.89	0.03	0.942	Hainan	0.917	0.076	0.87	Hainan

Confusion matrix				Confusion matrix			
a	b	←classified as		a	b	←classified as	
191	6	a = Yunnan		182	15	a = Yunnan	
12	97	b = Hainan		9	100	b = Hainan	

## Discussion

The National Malaria Elimination Programme (NMEP) was launched in 2010 with the goal to eliminate malaria from all regions by 2015, except the border regions in Yunnan province, and to completely eliminate malaria all over China by 2020 [43]. To guide the NMEP, effective surveillance-response strategies must to be tailored for local populations. Understanding the population structure, genetic diversity, and transmission patterns of the *Plasmodium* parasite in endemic regions will provide important information to facilitate the successful implementation of this programme.

Currently, falciparum malaria has been eliminated in most provinces because of the large-scale control activities. However, Yunnan and Hainan provinces of Southern China remain to be major endemic regions [2, 3]. This study was the first to evaluate the population structure, genetic diversity, and transmission patterns of *P. falciparum* in South China using highly polymorphic neutral markers and samples of field isolates collected from 2004 to 2008. All 13 trinucleotide microsatellite markers were extremely polymorphic for both Yunnan and Hainan provinces. The genetic diversity observed in the two *P. falciparum* populations was slightly different. For Yunnan Province, the microsatellite markers revealed high variation, the mean *Na* was 11.692 ± 1.216, and the mean *He* was 0.766 ± 0.036. While, for Hainan province, the microsatellite markers showed intermediate variation, the mean *Na* was 6.923 ± 0.923, and the mean *He* was 0.677 ± 0.039. However, the genetic diversity of *plasmodium falciparum* in South China (0.642–0.726) was slightly higher than that reported in several studies in southeast Asian countries [20–22],such as Thailand (0.391–0.841), Myanmar (0.60–0.68), Philippines (0.39–0.60), and Malaysian Borneo (0.44–0.63), also based on assaying genetic diversity at microsatellite loci. Meanwhile, compared to these southeast Asian countries, the proportions of multiple genotype infection was ranged from 0 (LeD) to 23% (TC), were lower than that of Thailand (0–44%), and Malaysian Borneo (17–71%), and higher than that of Philippines (0–10.7%). According to previous similarly studies based on microsatellite loci, these findings could be a result of the levels of malaria endemicity, i.e., the levels of genetic diversity of the parasite populations are higher in high transmission areas than in low transmission areas [19, 20].

Despite the high levels of genetic diversity, a significant linkage disequilibrium was found for the *P. falciparum* populations from some regions of Yunnan and Hainan provinces. Deviate from the general pattern established by Anderson et al.: microsatellite data revealed a spectrum of population structures in a single parasite species, that is, a strong LD was observed for regions of low transmission, while a weak LD was observed for regions of high transmission [19]. The LD pattern exhibited by the samples from LZ, BN, DF, and SY was high population diversity with high LD, indicating a low recombination due to largely clonal transmission and limited opportunity for transmission. In contrast, LD for the samples from DH, TC, and LeD exhibited a pattern consistent with the above-mentioned pattern [19]. The samples from all three catchments showed high levels of genetic diversity and the lack of a significant LD, suggesting a negative correlation between the degree of LD and the transmission intensity of *P. falciparum* due to some epidemiological or ecological reason.

Generally, with regard to local *P. falciparum* populations, the proportion of multiple infections are related to the transmission intensity, which is an important parameter affecting *P. falciparum* mating because cross fertilization and recombination may occur between parasite gametes of either the same or different genotypes of the mosquito vector [22]. High level of transmission leading to cross fertilization and recombination, and causing multiple infection. Combined with previous studies [21–23, 44, 45], an inverse correlation between the proportion of multiple infection and the degree of LD is expected, and it is rapidly broken down by the recombination. In the present study, though there was a wide range of frequencies of multiple infections (0-23%) and a wide range of the degree of LD (0–0.117), a weak negative correlation was found, indicating that a variable frequence of recombination existing within different populations in southern China.

The level of genetic differentiation were moderate (Fst 0.039–0.214) between the seven populations in South China, and comparable with that of Philippines (Fst 0.096–0.144), and lower than that of Malaysian Borneo (0.038–0.376). High level of genetic differentiation of the populations in Malaysian Borneo are considered to be a result of fragmentation of the population structure owing to an effective malaria control and low migration rate of people in the endemic areas. Nowadays, due to the intense population movements within the country, there were high migration rate of people in the endemic areas, which may causing low level of genetic differentiation.

Population assignment is an important aspect of epidemiological studies. As demonstrated by the dendrogram based on the pairwise Fst matrix, the *P. falciparum* populations from South China were primarily structured into two major clusters by STRUCTURE software. The populations were found to be clearly clustered according to their geographic origin, except some isolates collected from LeD and SY, probably because of their limited sample size. Furthermore, the median-joining network diagram tree was used to clarify these findings. A further analysis using the STRUCTURE program at K = 3–5 revealed that, although microsatellite analysis can still discriminate *P. falciaprum* populations from Yunnan and Hainan, it can have limitations in distinguishing *P. falciparum* cases from each site. Among the Yunnan populations, we observed a large amount of mixing among the LZ, TC, and DH populations. This was probably due to the high level of transmission in China-Myanmar border [46], and it reflects the intense human movements within these three regions. In terms of geographic location, TC is not far away from DH, and both are located along the NuJiang river, while LZ is on the China–Myanmar border. The constant human movements in these three regions may cause the migration of diverse parasites into these regions, leading to a complex population structuring with more ancestor. It is difficult to distinguish *P. falciparum* cases using only microsatellite markers. However, the *P. falciparum* population from BN, which is also located in Yunnan province but far away from TC and DH, was quiet different from that from the other three regions and was relatively simple. This could be due to the limited human movements. For the Hainan populations, especially in DF population, the *P. falciparum* isolates collected from 2004 to 2007 seems have a common ancestor with LeD and SY. While, the isolates collected in 2008 may have a different ancestor. The analysis results of isolates collected from different years, suggesting another outbreak of falciparum malaria for unknown reasons (i.e. the efforts on local malaria control or a significant migration of labourers having worked in endemic areas). Nevertheless, despite the limitations in distinguishing *P. falciparum* cases from each site, microsatellite-based analysis with the STRUCTURE and Network programs confirmed the presence of two subgroups in Southern China, namely Yunnan and Hainan.

Further statistical analysis to identify effective marker sets for discriminating the origin of *P. falciparum* isolates from South China was performed. Two marker sets containing three microsatellite loci were selected and used to establish a stepwise classification, which correctly classified more than 92% isolates. The results clearly demonstrate the feasibility of accurate classification of individual isolates from a geographic region and the effectiveness of the selected marker sets as applicable tools for tracing the origin of falciparum malaria outbreaks in the future.

## Conclusions

This is the first study to report the genetic diversity, transmission patterns, and population structure of *P. falciparum* parasites in Southern China using 13 highly polymorphic microsatellite loci. The results showed a high level of genetic diversity co-occurring with significant multilocus LD. In addition, the parasite populations were clustered into two subgroups according to their geographic locations, and the intensity of human movements could affect population assignment. Finally, two marker sets were selected to be used for tracing the origin of falciparum malaria outbreaks in the future. These findings provide important information for understanding the population structure of *P. falciparum* in Southern China and will allow investigators to focus their attention on very small geographic regions and rapidly formulate appropriate therapeutic strategies, which will eventually control and eliminate this disease completely.

## Additional files

Additional file 1: The full genotype data for all isolates. Individual genotypes with one row per isolate and one column per locus. Additional file 2: Allele frequencies at 13 microsatellite loci of the seven *Plasmodium falciparum* populations in the South China. Additional file 3: Summary of microsatellite markers and the genetic diversity (number of alleles per locus) of *Plasmodium falciparum* in southern China. Additional file 4: Bayesian cluster analysis using the STRUCTURE program: results for K = 3-5. A) Estimated population assignments of *Plasmodium falciparum* based on Bayesian cluster analysis of 13 microsatellite loci from 306 individuals at K=3-5, where each colour corresponds to a suggested cluster and each individual is represented by a single line. B) Population structure of *Plasmodium falciparum* plotted in multiple lines according to geographic origin at K=5. Isolates 1-197 were collected in Yunnan-Myanmar Region: 1-58 in MD, mainly 2006; 59-99 in DH, 2007; 100-171 in TC, 2006; 172-197 in BN, 2006. Isolates 198-306 were collected in Hainan Province: 198-213 in DF, 2004; 214-242 in DF, 2007; 243-280 in DF, 2008; 281-295 in LeD, 2004; 296-306 in SY, 2007. Additional file 5: Selected marker sets to differentiate *Plasmodium falciparum* isolates between Yunnan and Hainan provinces with high accuracy.
