# Supplementary material for: Evaluation of the population structure and genetic diversity of Plasmodium falciparum in southern China
Source: Malar J. 2015 Jul 22;14:283. doi: 10.1186/s12936-015-0786-0 (PMC4509482; doi:10.1186/s12936-015-0786-0)
Supplement: Additional file 2: — Allele frequencies at 13 microsatellite loci of the seven Plasmodium falciparum populations in the South China. [file 12936_2015_786_MOESM2_ESM.pdf]

Additional file 2. Allele frequencies at 13 microsatellite loci of the seven *plasmodium falciparum* populations in the South China.

**MS loci** (13)	**No.of alleles**	**Alleles** （bp）	**Yunnan**				**Hainan**			**Private alleles**
			**Lazan** (n=56)	**Dehong** (n=40)	**Tengchong** (n=70)	**Banna** (n=26)	**Dongfang** (n=83)	**Ledong** (n=15)	**Sanya** (n=11)	
**TA1**	1	134	…	…	…	…	0.012	…	…	Dongfang
	2	152	0.018	…	0.029	…	…	…	…	
	3	155	0.125	…	0.086	…	…	…	…	
	4	158	0.107	0.075	0.271	0.038	…	…	0.091	
	5	161	0.250	0.250	0.200	0.308	0.072	0.133	0.091	
	6	164	0.321	0.275	0.143	0.423	0.373	0.400	0.091	
	7	167	0.107	0.200	0.143	0.115	0.410	0.333	…	
	8	170	0.071	0.100	0.057	0.077	0.108	…	0.091	
	9	173	…	0.050	…	0.038	0.012	0.067	0.545	
	10	176	…	…	…	…	0.012	0.067	…	
	11	179	…	0.025	…	…	…	…	0.091	
	12	185	…	…	0.014	…	…	…	…	Tengchong
	13	188	…	…	0.057	…	…	…	…	Tengchong
	14	191	…	0.025	…	…	…	…	…	Dehong
**Polyα**	1	140	0.018	…	…	…	…	…	…	Lazan
	2	143	0.054	…	0.029	…	…	…	…	
	3	146	…	0.050	0.057	…	0.048	0.200	…	
	4	149	0.054	0.075	0.014	0.077	…	…	…	
	5	152	0.018	0.025	0.043	0.038	0.373	…	…	
	6	155	0.089	0.225	0.129	0.269	0.229	0.133	0.636	
	7	158	0.179	0.175	0.086	0.038	0.024	…	…	
	8	161	0.107	0.050	0.086	…	0.012	0.067	0.273	
	9	164	0.036	0.050	0.100	…	0.084	…	…	
	10	167	0.143	0.025	0.086	0.038	0.133	0.133	…	
	11	170	0.179	0.225	0.229	…	0.072	0.467	0.091	

MS loci (13)	No.of alleles	Alleles （bp）	Yunnan				Hainan			Private alleles
			Lazan (n=56)	Dehong (n=40)	Tengchong (n=70)	Banna (n=26)	Dongfang (n=83)	Ledong (n=15)	Sanya (n=11)	
**Polyα**	12	173	0.018	…	0.043	0.500	…	…	…	
	13	176	0.089	0.075	0.071	0.038	0.024	…	…	
	14	182	…	0.025	…	…	…	…	…	Dehong
	15	185	0.018	…	…	…	…	…	…	Lazan
	16	188	…	…	0.029	…	…	…	…	Tengchong
**PfPk2**	1	144	…	…	…	0.038	…	…	…	Banna
	2	153	…	…	…	0.115	…	…	…	Banna
	3	156	…	…	…	0.192	…	…	…	Banna
	4	159	0.018	0.025	0.014	0.077	0.060	…	…	
	5	162	0.054	0.150	0.057	0.192	0.036	…	…	
	6	165	0.054	0.175	0.100	0.115	0.048	…	0.182	
	7	168	0.125	0.275	0.143	…	0.072	0.133	0.182	
	8	171	0.375	0.150	0.243	0.038	0.024	0.467	0.273	
	9	174	0.161	0.150	0.200	0.192	0.060	0.133	…	
	10	177	0.071	0.025	0.100	0.038	0.398	…	…	
	11	180	0.018	…	0.043	…	0.120	…	…	
	12	183	0.018	…	0.014	…	…	0.200	0.091	
	13	186	0.036	…	0.014	…	0.024	…	0.091	
	14	189	0.036	…	0.029	…	0.108	…	…	
	15	192	…	…	0.014	…	0.048	…	…	
	16	195	0.018	0.025	0.014	…	…	…	…	
	17	198	0.018	0.000	0.014	…	…	…	…	
	18	201	…	0.025	…	…	…	…	…	Dehong
	19	204	…	…	…	…	…	…	0.182	Sanya
	20	207	…	…	…	…	…	0.067	…	Ledong
**TA81**	1	105	…	0.025	…	…	…	…	…	Dehong
	2	108	…	…	0.029	…	…	…	…	Tengchong

MS loci (13)	No.of alleles	Alleles （bp）	Yunnan				Hainan			Private alleles
			Lazan (n=56)	Dehong (n=40)	Tengchong (n=70)	Banna (n=26)	Dongfang (n=83)	Ledong (n=15)	Sanya (n=11)	
**TA81**	3	111	0.071	0.100	0.057	…	…	0.067	…	
	4	114	0.321	0.175	0.200	0.077	0.072	0.200	0.182	
	5	117	0.286	0.150	0.157	0.115	0.434	0.267	0.545	
	6	120	0.054	0.225	0.014	0.385	0.265	0.067	…	
	7	123	0.143	0.150	0.214	0.385	0.133	0.200	0.182	
	8	126	0.018	0.150	0.157	…	0.036	…	0.091	
	9	129	0.071	…	0.114	0.038	0.048	0.200	…	
	10	132	0.036	…	0.043	…	0.012	…	…	
	11	135	…	0.025	…	…	…	…	…	Dehong
	12	141	…	…	0.014	…	…	…	…	Tengchong
**TA109**	1	137	…	…	…	0.038	…	…	…	Banna
	2	152	0.018	0.025	…	…	0.301	0.600	0.455	
	3	155	0.714	0.450	0.843	…	0.241	0.333	0.545	
	4	158	0.179	0.400	0.043	0.538	0.289	…	…	
	5	161	…	…	…	0.192	0.169	…	…	
	6	164	…	…	…	…	…	0.067	…	Ledong
	7	167	0.071	0.050	0.100	…	…	…	…	
	8	170	0.018	0.025	0.014	0.192	…	…	…	
	9	179	…	0.025	…	…	…	…	…	Dehong
	10	185	…	0.025	…	…	…	…	…	Dehong
	11	188	…	…	…	0.038	…	…	…	Banna
**TA42**	1	172	…	0.025	…	…	…	…	…	Dehong
	2	175	0.018	…	0.014	…	…	…	…	
	3	178	0.411	0.875	0.871	0.077	0.157	0.133	0.091	
	4	181	0.536	0.025	0.043	0.231	0.735	0.533	0.727	
	5	184	…	…	…	0.385	0.024	0.200	…	
	6	187	…	…	…	0.038	…	…	…	Banna

MS loci (13)	No.of alleles	Alleles （bp）	Yunnan				Hainan			Private alleles
			Lazan (n=56)	Dehong (n=40)	Tengchong (n=70)	Banna (n=26)	Dongfang (n=83)	Ledong (n=15)	Sanya (n=11)	
**TA42**	7	193	…	…	0.014	…	…	…	…	Tengchong
	8	235	…	0.025	…	0.038	…	…	…	
	9	238	0.018	0.025	0.057	…	…	…	…	
	10	241	0.018	0.025	…	0.231	0.072	0.067	0.091	
	11	244	…	…	…	…	0.012	0.067	0.091	
**TA60**	1	64	0.018	…	…	…	…	…	…	Lazan
	2	73	0.036	0.075	0.057	0.231	…	…	…	
	3	76	0.107	0.150	0.129	0.154	…	…	…	
	4	79	0.143	0.225	0.157	0.077	0.193	0.200	0.455	
	5	82	0.411	0.350	0.386	0.423	0.723	0.600	0.455	
	6	85	0.232	0.050	0.157	0.077	0.084	0.133	…	
	7	88	…	0.050	0.057	0.038	…	0.067	0.091	
	8	91	0.036	0.075	0.057	…	…	…	…	
	9	94	0.018	0.025	…	…	…	…	…	
**TA87**	1	84	…	0.025	0.014	…	…	…	…	
	2	87	0.018	0.025	0.057	…	…	…	…	
	3	90	0.036	0.025	0.014	…	…	…	…	
	4	93	0.143	0.075	…	…	…	…	…	
	5	96	0.232	0.250	0.114	0.077	0.024	…	…	
	6	99	0.196	0.125	0.143	0.038	0.193	0.733	0.545	
	7	102	0.161	0.300	0.243	0.385	0.494	0.133	0.091	
	8	105	0.179	0.125	0.329	0.231	0.277	0.133	0.364	
	9	108	…	0.025	0.043	0.077	0.012	…	…	
	10	111	0.018	…	0.014	0.077	…	…	…	
	11	114	…	0.025	0.014	0.115	…	…	…	
	12	120	0.018	…	0.014	…	…	…	…	
**ARA2**	1	61	…	0.050	…	…	…	…	…	Dehong

MS loci (13)	No.of alleles	Alleles （bp）	Yunnan				Hainan			Private alleles
			Lazan (n=56)	Dehong (n=40)	Tengchong (n=70)	Banna (n=26)	Dongfang (n=83)	Ledong (n=15)	Sanya (n=11)	
**ARA2**	2	64	0.018	…	0.029	…	0.012	…	…	
	3	67	0.018	0.025	0.043	0.231	0.108	…	…	
	4	70	0.375	0.100	0.214	0.231	0.410	0.267	0.545	
	5	73	0.107	0.150	0.100	0.308	0.084	0.267	0.182	
	6	76	0.357	0.425	0.414	0.115	0.120	0.333	0.182	
	7	79	0.071	0.200	0.071	…	0.096	0.067	…	
	8	82	…	0.025	0.086	0.115	0.169	0.067	0.091	
	9	85	0.054	0.025	0.029	…	…	…	…	
	10	97	…	…	0.014	…	…	…	…	Tengchong
**2490**	1	74	…	0.050	0.029	…	…	…	…	
	2	77	0.018	…	0.043	…	0.012	…	0.545	
	3	80	0.232	0.600	0.529	0.846	0.518	0.067	0.091	
	4	83	0.446	0.350	0.357	0.115	0.410	0.667	0.273	
	5	86	0.304	…	0.043	0.038	0.060	0.267	0.091	
**Pfg377**	1	80	…	…	0.014	…	…	…	…	Tengchong
	2	86	0.018	0.075	…	…	…	…	…	
	3	89	0.054	0.650	0.171	…	0.349	0.533	0.273	
	4	92	0.786	0.250	0.714	0.038	0.651	0.467	0.636	
	5	95	0.143	0.025	0.100	0.769	…	…	0.091	
	6	98	…	…	…	0.192	…	…	…	Banna
**B5M2**	1	130	…	0.025	…	…	…	…	…	Dehong
	2	136	0.036	0.075	0.029	…	…	…	…	
	3	139	0.214	0.350	0.314	0.077	0.036	…	…	
	4	142	0.107	0.025	0.029	0.231	0.675	0.400	0.636	
	5	145	0.036	0.075	0.057	0.308	0.229	0.467	…	
	6	148	…	0.050	0.043	0.115	0.060	0.133	0.091	
	7	151	0.071	0.050	…	…	…	…	0.273	

MS loci (13)	No.of alleles	Alleles （bp）	Yunnan				Hainan			Private alleles
			Lazan (n=56)	Dehong (n=40)	Tengchong (n=70)	Banna (n=26)	Dongfang (n=83)	Ledong (n=15)	Sanya (n=11)	
**B5M2**	8	154	0.089	0.275	0.057	0.192	…	…	…	
	9	157	0.232	0.025	0.371	0.077	…	…	…	
	10	160	0.107	0.025	0.043	…	…	…	…	
	11	163	0.107	0.025	0.057	…	…	…	…	
**C1M8**	1	128	…	…	…	0.077	…	…	…	Banna
	2	131	…	…	…	0.038	0.036	…	…	
	3	143	0.036	…	0.014	…	…	…	…	
	4	149	0.036	…	0.043	…	…	…	…	
	5	152	0.054	0.125	0.057	…	…	…	…	
	6	155	0.036	0.025	0.100	0.192	…	…	…	
	7	158	0.143	0.050	0.043	…	…	…	…	
	8	161	0.268	0.100	0.143	0.115	0.024	0.067	0.091	
	9	164	0.089	0.150	0.029	0.038	0.084	0.400	0.273	
	10	167	0.054	0.075	0.157	0.346	0.157	…	0.091	
	11	170	0.054	0.250	0.086	0.038	…	0.067	…	
	12	173	0.018	0.025	0.029	…	0.157	…	0.091	
	13	176	0.054	0.050	0.129	0.115	0.386	0.067	0.364	
	14	179	0.018	0.025	0.057	…	0.084	…	…	
	15	182	0.036	…	0.057	…	…	…	0.091	
	16	185	0.018	0.050	…	…	…	…	…	
	17	188	0.018	0.025	0.014	0.038	…	0.133	…	
	18	191	0.054	…	0.029	…	0.036	0.200	…	
	19	194	0.018	…	…	…	0.036	0.067	…	
	20	197	…	0.050	0.014	…	…	…	…	
