# Supplementary material for: Evaluation of the population structure and genetic diversity of Plasmodium falciparum in southern China
Source: Malar J. 2015 Jul 22;14:283. doi: 10.1186/s12936-015-0786-0 (PMC4509482; doi:10.1186/s12936-015-0786-0)
Supplement: Additional file 3: — Summary of microsatellite markers and the genetic diversity (number of alleles per locus) of Plasmodium falciparum in southern China. [file 12936_2015_786_MOESM3_ESM.pdf]

Additional file 3: Summary of microsatellite markers and the genetic diversity of *Plasmodium falciparum* in southern China

**Locus**	GeneBank accession number	Chromosome	Allele size(bp)	Number of alleles	He
**TA1**	AF010507	6	134-191	14	0.817
**Polyα**	L18785	4	140-188	16	0.892
**PfPK2**	X63648	12	144-207	20	0.891
**TA81**	AF010510	5	105-141	12	0.831
**TA109**	AF010508	6	137-188	11	0.683
**TA42**	AF010543	5	172-244	11	0.636
**TA60**	AF010556	13	64-94	9	0.697
**TA87**	AF010571	6	84-120	12	0.796
**ARA2**	X17848	11	61-97	10	0.796
**2490**	T02490	10	74-86	5	0.629
**Pfg377**	L04161	12	80-98	6	0.58
**B5M2**	G44403	7	130-163	11	0.841
**C1M8**	G38013	1	128-197	20	0.91
**Mean±SE**				12.154±1.285	0.769±0.031
