# Supplementary material for: Evaluation of the population structure and genetic diversity of Plasmodium falciparum in southern China
Source: Malar J. 2015 Jul 22;14:283. doi: 10.1186/s12936-015-0786-0 (PMC4509482; doi:10.1186/s12936-015-0786-0)

Additional file 4 Bayesian cluster analysis using the STRUCTURE program: results for K = 3-5. (A) Estimated population assignments of *plasmodium falciparum* based on Bayesian cluster analysis of 13 microsatellite loci from 306 individuals at K=3-5, where each color corresponds to a suggested cluster and each individual is represented by a single line. (B) Population structure of *plasmodium falciparum* plotted in multiple lines according to geographic origin at K=5. Isolates 1-197 were collected in Yunnan-Myanrmar Region: 1-58 in LZ, mainly 2006; 59-99 in DH, 2007; 100-171 in TC, 2006; 172-197 in BN, 2006. Isolates 198-306 were collected in Hainan Province: 198-213 in DF, 2004; 214-242 in DF, 2007; 243-280 in DF, 2008; 281-295 in LeD, 2004; 296-306 in SY, 2007.

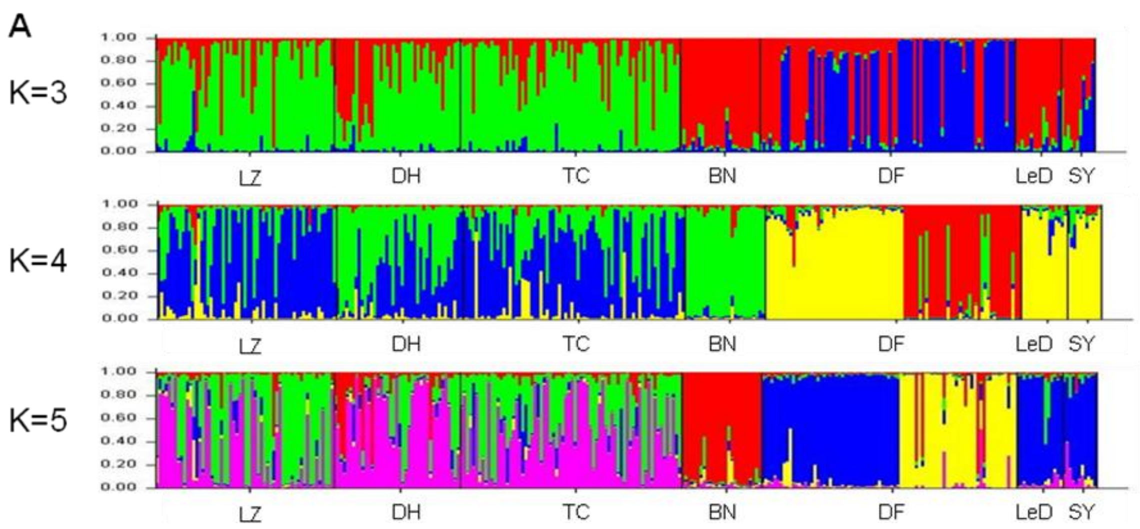

B

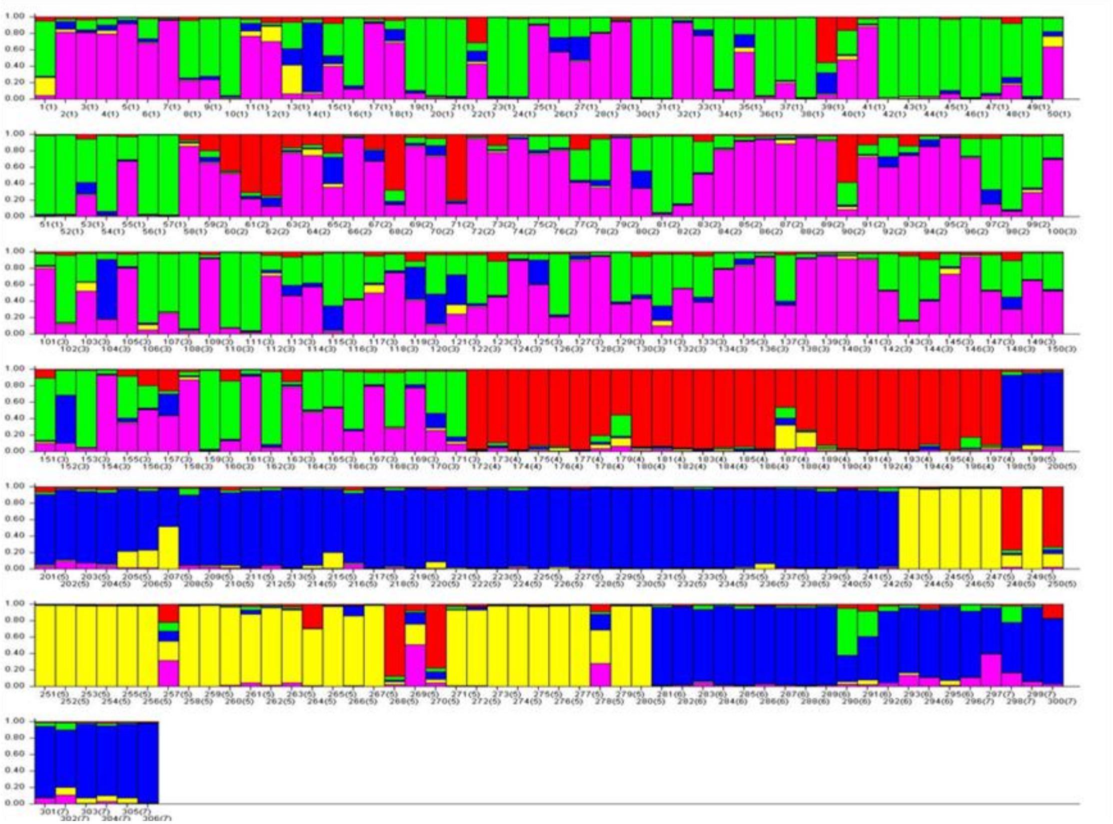

Supplement: Additional file 4: — Bayesian cluster analysis using the STRUCTURE program: results for K = 3-5. A) Estimated population assignments of Plasmodium falciparum based on Bayesian cluster analysis of 13 microsatellite loci from 306 individuals at K=3-5, where each colour corresponds to a suggested cluster and each individual is represented by a single line. B) Population structure of Plasmodium falciparum plotted in multiple lines according to geographic origin at K=5. Isolates 1-197 were collected in Yunnan-Myanmar Region: 1-58 in MD, mainly 2006; 59-99 in DH, 2007; 100-171 in TC, 2006; 172-197 in BN, 2006. Isolates 198-306 were collected in Hainan Province: 198-213 in DF, 2004; 214-242 in DF, 2007; 243-280 in DF, 2008; 281-295 in LeD, 2004; 296-306 in SY, 2007. [file 12936_2015_786_MOESM4_ESM.pdf]
