# Supplementary material for: Evaluation of the population structure and genetic diversity of Plasmodium falciparum in southern China
Source: Malar J. 2015 Jul 22;14:283. doi: 10.1186/s12936-015-0786-0 (PMC4509482; doi:10.1186/s12936-015-0786-0)
Supplement: Additional file 5: — Selected marker sets to differentiate Plasmodium falciparum isolates between Yunnan and Hainan provinces with high accuracy. [file 12936_2015_786_MOESM5_ESM.pdf]

Additional file 5: Selected marker sets to differentiate *P.falciparum* isolates between Yunnan and Hainan provinces with high accuracy

Marker set	Correctly Classified Instances （%）	Class	
		Yunnan (%)	Hainan (%)
B5M2	84.314	82.7	87.2
TA1,B5M2	89.543	92.4	84.4
Pfg377,B5M2	90.523	90.4	90.8
ARA2,B5M2	85.621	87.8	81.7
TA81,B5M2	80.719	86.3	83.5
TA60,B5M2	89.869	89.8	89.9
TA42,Pfg377	82.026	82.7	80.7
TA42,B5M2	87.909	88.8	86.2
TA1,Pfg377,B5M2	94.118	97	89
TA109,TA42,B5M2	91.177	94.4	85.3
TA109,TA87,Pfg377	87.255	92.4	84.4
TA109,Pfg377,B5M2	90.196	94.4	82.6
TA42,TA60,B5M2	88.562	92.9	80.7
TA42,TA87,B5M2	89.216	92.9	82.6

Marker set	Correctly Classified Instances （%）	Class	
		Yunnan (%)	Hainan (%)
TA42,Pfg377,B5M2	90.85	92.9	87.2
TA60,TA87,B5M2	90.523	93.4	85.3
TA60,2490,B5M2	88.562	91.9	82.6
TA60,Pfg377,B5M2	91.83	93.4	89
TA87,Pfg377,B5M2	91.503	92.9	89
2490,Pfg377,B5M2	92.157	92.4	91.7
TA1,TA109,Pfg377,B5M2	90.85	98	78
TA1,TA60,Pfg377,B5M2	90.523	97.5	78
TA1,TA87,Pfg377,B5M2	90.523	97.5	78
TA1,2490,Pfg377,B5M2	89.869	97	78.9
TA109,TA42,Pfg377,B5M2	90.523	97	78.9
TA109,TA87,Pfg377,B5M2	91.177	97.5	79.8
TA109,ARA2,Pfg377,B5M2	90.196	97.5	78
TA109,2490,Pfg377,B5M2	91.503	98	80.7
